# Development of the first marmoset-specific DNA microarray (EUMAMA): a new genetic tool for large-scale expression profiling in a non-human primate

**DOI:** 10.1186/1471-2164-8-190

**Published:** 2007-06-25

**Authors:** Nicole A Datson, Maarten C Morsink, Srebrena Atanasova, Victor W Armstrong, Hans Zischler, Christina Schlumbohm, Bas E Dutilh, Martijn A Huynen, Brigitte Waegele, Andreas Ruepp, E Ronald de Kloet, Eberhard Fuchs

**Affiliations:** 1 Division of Medical Pharmacology, Leiden/Amsterdam Center for Drug Research and Leiden University Medical Center, The Netherlands; 2 Department of Clinical Chemistry, Georg-August University, Goettingen, Germany; 3 Institute of Anthropology, University of Mainz, Mainz, Germany; 4 Clinical Neurobiology Laboratory, German Primate Center, Göttingen, Germany; 5 Center for Molecular and Biomolecular Informatics/Nijmegen Center for Molecular Life Sciences, Radboud University Nijmegen, Geert Grooteplein 28, 6525 GA, Nijmegen, The Netherlands; 6 Institute for Bioinformatics, GSF – National Research Center for Environment and Health, Ingolstaedter Landstrasse 1, Germany

## Abstract

**Background:**

The common marmoset monkey (*Callithrix jacchus*), a small non-endangered New World primate native to eastern Brazil, is becoming increasingly used as a non-human primate model in biomedical research, drug development and safety assessment. In contrast to the growing interest for the marmoset as an animal model, the molecular tools for genetic analysis are extremely limited.

**Results:**

Here we report the development of the first marmoset-specific oligonucleotide microarray (EUMAMA) containing probe sets targeting 1541 different marmoset transcripts expressed in hippocampus. These 1541 transcripts represent a wide variety of different functional gene classes. Hybridisation of the marmoset microarray with labelled RNA from hippocampus, cortex and a panel of 7 different peripheral tissues resulted in high detection rates of 85% in the neuronal tissues and on average 70% in the non-neuronal tissues. The expression profiles of the 2 neuronal tissues, hippocampus and cortex, were highly similar, as indicated by a correlation coefficient of 0.96. Several transcripts with a tissue-specific pattern of expression were identified. Besides the marmoset microarray we have generated 3215 ESTs derived from marmoset hippocampus, which have been annotated and submitted to GenBank [GenBank: EF214838 – EF215447, EH380242 – EH382846].

**Conclusion:**

We have generated the first marmoset-specific DNA microarray and demonstrated its use to characterise large-scale gene expression profiles of hippocampus but also of other neuronal and non-neuronal tissues. In addition, we have generated a large collection of ESTs of marmoset origin, which are now available in the public domain. These new tools will facilitate molecular genetic research into this non-human primate animal model.

## Background

The common marmoset monkey (*Callithrix jacchus*), a small non-endangered New World primate native to eastern Brazil, is becoming increasingly used as a non-human primate model in biomedical research, drug development and safety assessment. Not only their close genetic, physiological and metabolic similarity to humans but also several unique physiological differences between Old World and New World primates, underlie its widespread use as an animal model in studies involving aspects of infectious disease, stem cell research, neural and cognitive sciences, toxicology and drug development and reproductive biology. Moreover, its availability, size, ease of breeding in laboratory conditions and its clear advantages over other non-human primates in terms of animal welfare, costs and practicality contribute to its popularity as an attractive alternative to other non-human primate species such as the rhesus macaque [1]. In contrast to the growing interest for the marmoset as an animal model, the molecular tools for genetic analysis are extremely limited.

Although a Marmoset Genome Project was initiated in 2004 and is ongoing [2], today there are still only very few mRNA sequences of marmoset origin available in the public domain. To allow gene expression profiling of different marmoset organs and delineation of complex biological pathways, we have developed a marmoset-specific DNA microarray, called EUMAMA (EUropean MArmoset MicroArray) starting from large scale EST sequencing of marmoset transcripts to generate the required sequence information.

The marmoset array is an initiative within a European Commission-funded consortium aimed at investigating the long term effects of prenatal exposure to glucocorticoids [3] (hormones secreted by the adrenal cortex in response to stress) in a non-human primate model. The working hypothesis of this consortium is that excessive foetal exposure to glucocorticoid hormones (GCs) exerts organisational effects on the development of a variety of target tissues resulting in altered gene expression patterns that may persist throughout life. These 'programming' effects are believed to shape vulnerability to a variety of adult diseases including heart disease, diabetes, kidney failure and brain disorders [4-8]. Since the effects of prenatal GC exposure are so widespread, we chose to initially focus on the effects in the brain by using hippocampal RNA as a source to generate EST sequences on the marmoset array. The hippocampus is a brain structure involved in learning and memory processes, mood and regulation of the stress response via the hypothalamic-pituitary-adrenal axis. GCs are essential for normal hippocampal development, exerting a wide spectrum of effects, resulting in altered structural plasticity and function [9-12]. Therefore, the obvious applicability of the marmoset microarray is to study neuronal gene expression. However, we show here that the microarray can also be used to generate expression profiles in a wide variety of non-neuronal marmoset tissues.

Besides the marmoset microarray we have generated 3215 ESTs derived from marmoset hippocampus, which have been functionally annotated and submitted to GenBank [GenBank: EF214838 – EF215447, EH380242 – EH382846]. These new tools will facilitate molecular genetic research into this non-human primate animal model.

## Results

### Mapping, annotation and submission of sequences to GenBank

**Figure 1:**
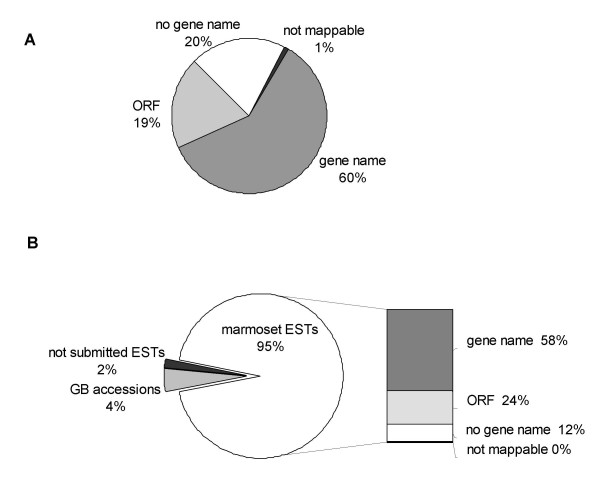
**Pie charts. A: Composition of 3215 marmoset ESTs**. Pie chart indicating the composition of the 3215 marmoset ESTs derived from a normalized hippocampal cDNA library. After mapping and annotation, a total of 1932 (60%) ESTs were assigned a gene name, 610 (19%) contained a partial ORF, 642 (20%) were mappable to a genome but could not be assigned a gene name and 31 (1%) could not be mapped. **B: Origin of marmoset sequences**. Pie chart indicating the origin of the marmoset sequences represented on the marmoset microarray. Of the in total 1541 marmoset transcripts represented on the array the majority (1445 = 95%) were derived from the set of 3215 marmoset ESTs submitted to GenBank. The remaining 5% consisted of 68 pre-existing marmoset sequences already present in GenBank and 28 ESTs from the hippocampal cDNA library that were not submitted to GenBank. The 1445 submitted ESTs could be subdivided into a group of 886 (58%) that were assigned a gene name, 364 (24%) with a (partial) ORF, 188 (12%) that were mappable but without a gene name or an ORF and 7 (0%) that were not mappable.

A total of 3441 high quality sequences (Phred score: Q15>600; 87.5% of the sequences also passed the threshold of Q20>600 bp; the overall minimal Q20 score was 429 bp) were selected for annotation [13]. After removal of redundant sequences, 3215 high quality unique 3' EST sequences remained which were trimmed to an average length of 678 bases. Annotation of the EST dataset to the RefSeq dataset of human genes allowed unambiguous assignment of gene names for 2250 sequences. A remaining 292 sequences could also be confidently assigned with gene names by mapping to the human genome or cross-species mapping. Of these 2542 (2250 + 292) sequences assigned with a gene name, 610 (19%) sequences could also be annotated with an open reading frame (ORF) (Figure 1A). These ORF-containing sequences were submitted to GenBank [GenBank: EF214838 – EF215447]. The majority of the remaining 673 sequences without an assigned gene name consisted of a group of 642 (20%) which could not be matched to a known mRNA but were mappable on the genome and 31 (1%) which could not mapped to RefSeq genes nor to a mammalian genome (Figure 1A). The latter 31 genes most likely represent novel genes that may be uniquely expressed in the marmoset hippocampus. All 2605 (81%) EST sequences without an ORF were submitted to dbEST [GenBank: EH380242 – EH382846].

### Gene selection and construction of the marmoset microarray

A subset of 1448 of the annotated 3'ESTs were selected by the EUPEAH consortium to be represented on the marmoset array, supplemented with 68 marmoset cDNA sequences available in GenBank. We selected the GeneChip^® ^CustomExpress^® ^Array Program of Affymetrix for design and manufacturing of the marmoset array (format 100–2187 containing approx. 1,600 gene sequences at 11 probe pairs per probe set). This choice was based on the previous experience of Affymetrix working with consortia to design specific dedicated arrays but also due to the advantages of standardisation of the hybridisation and data extraction procedure. The sequence data was submitted to the Affymetrix Chip Design Group and used to design 11 perfect match and mismatch 25-mer oligonucleotides per sequence according to the standard algorithms used in Affymetrix commercially available catalogue arrays. The most optimal probes sets per sequence were selected for in situ synthesis on the marmoset array. Each unique transcript was represented by a single probe set, except for 11 transcripts where there was no obvious ideal probe set. In these 11 cases 2 different probes sets were synthesized in situ representing different nucleotides of the same transcript. As controls the standard Affymetrix control set present on all Affymetrix expression arrays, consisting of quality control and alignment controls as well as target preparation and hybridisation controls, was tiled. Since we did not have a set of full-length housekeeping control genes of the marmoset for assessment of sample quality, scaling and/or normalization of arrays, the species-specific control sets of rhesus monkey and human were tiled. In addition, both 5' and 3' sequences of 11 marmoset genes abundantly expressed in hippocampus were included as controls for assessing the overall labeling efficiency and quality of biotin-labeled target sample (Table 1).

**Table 1:** Marmoset-specific controls on the array. An overview of the marmoset-specific controls present on the array. These controls represent transcripts abundantly expressed in hippocampus and probe sets from both the 3' and the 5' part of the transcript were selected for tiling on the array, as indicated by the probe set ID.

**Probe Set ID**	**Gene Symbol**	**Gene Title**
ACTB-5_at	ACTB	beta actin
ACTB-M_x_at	ACTB	beta actin
ADD1-3_at	ADD1	adducin 1 (alpha)
ADD1-5_at	ADD1	adducin 1 (alpha)
ALDH9A1-3_at	ALDH9A1	aldehyde dehydrogenase 9 family, member A1
ALDH9A1-5_s_at	ALDH9A1	aldehyde dehydrogenase 9 family, member A1
CAMK1G-3_at	CAMK1G	calcium calmodulin-dependent protein kinase IG
CAMK1G-5_at	CAMK1G	calcium calmodulin-dependent protein kinase IG
CCT8-3_at	CCT8	chaperonin containing TCP1, subunit 8 (theta)
CCT8-5_s_at	CCT8	chaperonin containing TCP1, subunit 8 (theta)
HDAC3-3_at	HDAC3	histone deacetylase 3
HDAC3-5_s_at	HDAC3	histone deacetylase 3
LPL-3_at	LPL	lipoprotein lipase
LPL-5_at	LPL	lipoprotein lipase
LRPAP1-3_at	LRPAP1	low density lipoprotein receptor-related protein associated protein 1
LRPAP1-5_s_at	LRPAP1	low density lipoprotein receptor-related protein associated protein 1
PLD3-3_x_at	PLD3	phospholipase D3
PLD3-5_s_at	PLD3	phospholipase D3
UBE2G2-3_at	UBE2G2	ubiquitin-conjugating enzyme E2G 2 (UBC7 homolog, yeast)
UBE2G2-5_at	UBE2G2	ubiquitin-conjugating enzyme E2G 2 (UBC7 homolog, yeast)
ZAK-3_s_at	ZAK	sterile alpha motif and leucine zipper containing kinase AZK
ZAK-5_s_at	ZAK	sterile alpha motif and leucine zipper containing kinase AZK

The final design of the marmoset microarray consisted of a total of 1649 probe sets of which 97 control probe sets and 1552 probe sets representing 1541 different marmoset transcripts. A total of 71 probe sets were derived from 68 different sequences already available in GenBank, while the remaining 1481 probe sets were derived from 1473 of our proprietary sequences obtained by sequencing marmoset ESTs. The majority (1445) of these 1473 ESTs were derived from the set of 3215 ESTs that have been submitted to GenBank (Figure 1B). Another 28 ESTs had already been selected for representation on the marmoset array based on our initial annotation, but did not pass the criteria we applied for GenBank submission.

The majority of the ESTs represented on the marmoset array assigned with a gene symbol (in total 1251) could be classified according to gene ontology based on biological function (1034) or molecular process (1106), representing a wide variety of different functional classes (Additional file 1).

### Expression profiling of hippocampus and several non-neuronal tissues

Detection rates, indicated by present calls, on RNA derived from hippocampus and cortex were high with 85% present calls. Even though the sequences represented on the marmoset GeneChip are of hippocampal origin, detection rates on RNA derived from various peripheral tissues, including kidney, liver, adipose tissue, aorta, skeletal muscle, ovary and testis, were also high. All peripheral tissues tested so far displayed approximately 70% present calls, with the exception of adipose tissue that had an overall level of detection of 78% (Table 2).

**Table 2:** Detection rates in different tissues on the marmoset array. The percentage present, marginal and absent calls obtained by hybridising the marmoset microarray with RNA from a panel of different tissues are indicated.

**Tissue**	**% present**	**% marginal**	**% absent**
Hippocampus	84.8	0.9	14.3
Cortex	84.2	1.2	14.6
Fat	78.4	1.1	20.5
Aorta	70.6	1.7	27.6
Ovary	70.2	1.4	28.3
Liver	69.5	1.3	29.2
Muscle	69.4	1	29.6
Kidney	69.3	1.3	29.4
Testis	68.1	1.5	30.4

**Figure 2:**
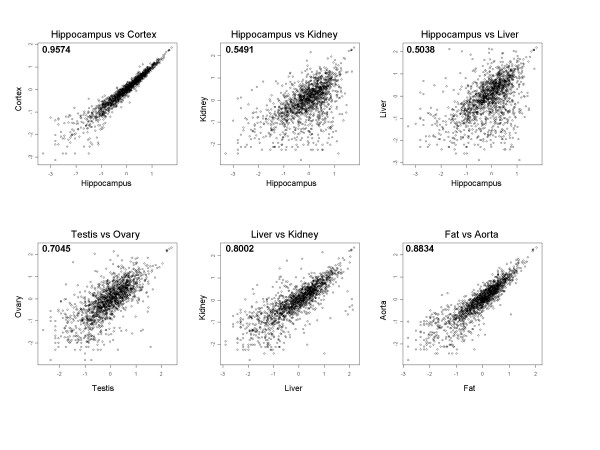
**Scatterplots comparing expression profiles of different tissues**. Scatterplots of log10-transformed signal intensities of several tissue comparisons showing the similarity of the expression profiles as indicated by the correlation coefficient.

Correlation coeffcients were calculated based on hybridisation signals on the marmoset microarray for all possible combinations of tissues (Table 3). The highest correlation coefficient (0.96) was obtained when comparing gene expression in both neuronal tissues, i.e. hippocampus and cortex, indicating a high degree of similarity in expression profiles. In contrast, comparing hippocampal gene expression to all peripheral tissues resulted in a severe drop in correlation coefficient (Figure 2), ranging from 0.69 in muscle to 0.50 in liver.

**Table 3:** Comparison of gene expression between different tissues as determined by hybridisation of the marmoset array. The Pearson product moment correlation coefficient, r, is indicated for the expression profiles of all possible combinations of tissues from our tissue panel.

	**Hippocampus**	**Cortex**	**Fat **	**Aorta**	**Ovary**	**Liver**	**Muscle**	**Kidney**	**Testis**
**Hippocampus**	**1**	0.95741	0.60714	0.56866	0.54152	0.50381	0.68915	0.54918	0.53917
**Cortex**		**1**	0.62146	0.58074	0.54732	0.51323	0.6862	0.74907	0.56225
**Fat **			**1**	0.8834	0.822	0.78857	0.7828	0.82475	0.68859
**Aorta**				**1**	0.81828	0.76857	0.78129	0.80429	0.67708
**Ovary**					**1**	0.76293	0.74486	0.80561	0.70452
**Liver**						**1**	0.7282	0.80027	0.65683
**Muscle**							**1**	0.74907	0.66837
**Kidney**								**1**	0.69856
**Testis**									**1**

Half of the probe sets of (767/1552 = 49.4%) were detectable (defined by a present call) in all tissues we tested so far, albeit in most cases at varying levels. Sorting of the expression profiles based on detection call allowed the identification of transcripts expressed exclusively in 1 of the 9 tissues tested. In liver, for example, we identified 7 probe sets with a present or marginal call that had absent calls in the other 8 tissues tested. Three of these 7 probes sets represented cytochrome p-450, enzymes that mediate the oxidative biotransformation of most drugs and are known to be predominantly expressed in liver [14]. Detection of 5 probe sets was restricted to the kidney, one of which represented (prepro)renin. Renin is part of the renin-angiotensin system known to play a major role in volume regulation and is expressed at high levels in the kidney [15]. Sperm protein 17 was exclusively expressed in testis. An explanation for the fact that we find a number of genes that appear to be exclusively expressed in other tissues than brain, while the origin of the ESTs is brain tissue, may be that these genes are predominantly derived from the list of 68 pre-existing GenBank sequences on the array, which are not necessarily expressed in brain. Indeed, this appears to be the case in many instances. For example, the 3 cytochrome p-450 probe sets exclusively expressed in liver are derived from pre-existing GenBank sequences, as well as 4 of the 5 kidney-specific probe sets (including renin) and 3 of the 4 testis-specific probe sets (including sperm protein 17). An additional explanation is that expression levels in brain may be too low to detect using microarrays, but were detectable in our normalised cDNA library of hippocampus.

Detection of a total of 82 probe sets was restricted to neuronal tissue (hippocampus and cortex) (Additional file 3). Among these 82 probe sets were several ion channels *(KCNAB3, KCNF1, KCNH3, SCN1a, CACNB1*), neurotransmitter receptors (*GABRA5, 5-HTR2C, GRIN1*), neural cell adhesion molecules (*NCAM, OPCML*) and diverse components involved in neurotransmitter secretion (*SYN2, SNPH*), axonogenesis (*SLITRK1*) and neurotrophic factor signalling (*NTRK2*). It must be noted that although the sequences have been annotated, the information is not that detailed that for each gene the exact isoform or splice variant is indicated. For example, a closer look at the data of CACNB1 indicates that only isoform 1 is targeted by our sequence, which indeed is expressed in brain, while isoform 2 is expressed in muscle. The users of the marmoset microarray have access to the exact probe set sequences as well as the EST sequences, allowing unambiguous determination of which part of the transcript is targeted and which splice variants or isoforms are detected.

Ninety probe sets were not detected in any of the tissues tested, which may be an intrinsic property of the selected probes or due to potential sequencing errors resulting in mismatches of the probe sets with the labeled mRNA (Additional file 3).

## Discussion

We have constructed a marmoset-specific oligonucleotide microarray representing 1541 different marmoset transcripts expressed in hippocampus. Although the sequences are primarily derived from a cDNA library of hippocampus, we have demonstrated that the marmoset array can also be efficiently used to characterise gene expression in other tissues of non-neuronal origin. The obtained detection rates are high, albeit somewhat lower in peripheral tissues than in neuronal tissue. The reproducibility of hybridisation is high, judged by the correlation (0.957) between both neuronal expression profiles (hippocampus and cortex). Moreover, the obtained tissue-specific patterns of expression are in line with what can be expected, with neuronal genes expressed exclusively in hippocampus and cortex, cytochrome p-450s in liver, preprorenin in kidney and sperm protein 17 in testis.

In the process of development of the marmoset microarray we have generated a set of 3215 marmoset 3' ESTs which have been submitted to GenBank. The number of marmoset sequences present today in publically available sequence databases including GenBank are 1433 (query "*callithrix jacchus*" at the National Center for Biotechnology Information [16]), which is a mix of working draft genomic sequence of the marmoset genome project, (partial) mRNA sequences and ESTs and contains several redundancies. Our contribution of 3215 different 3' ESTs is a substantial expansion of the number of marmoset sequences. Both the marmoset array and the generated ESTs therefore significantly expand the available molecular tools for genetic analysis in this non-human primate animal model.

To our knowledge EUMAMA is the first DNA microarray specific for the common marmoset. As is the case with all first-generation microarrays for a species, some day in the future our marmoset microarray is likely to become obsolete and be replaced by an array targeting a larger part of the transcriptome. Until there is a better replacement (e.g when the marmoset genome is sequenced) this microarray is the only molecular tool allowing analysis of gene expression of a reasonably comprehensive number of genes expressed in marmoset brain. The strength of this array is not only that it is species-specific, but also that there are corresponding marmoset sequences available for genetic follow-up of differentially expressed transcripts. So far the availability of microarrays specific for non-human primates is very limited. Oligonucleotide microarrays for the rhesus macaque (*Macaca mulatta*) have recently become commercially available at both Affymetrix and Agilent [17]. However, today expression profiling in non-human primates including chimpanzee (*Pan troglodytes*), orangutan (*Pongo pygmaeus*), African green monkey (*Chlorocebus aethiops*), common marmoset (*Callithrix jacchus*) and cynomolgus monkeys (*Macaca fascicularis*) is still performed using human arrays [18-23], despite the fact that sequence mismatches affect hybridisation intensity and likely result in a high level of false negatives or underrepresented expressed genes [18,24,25]. Interspecies use of microarrays is particularly problematic when using human microarrays to study gene expression in non-human primates that are more divergent to humans than the great apes. While chimpanzee or orangutan have an average nucleotide sequence divergence of 0.8–1% and 3% respectively [26-28], the average sequence divergence of human and rhesus macaque is approximately 5% [29,30] increasing to 11% when comparing the marmoset and human genome [31]. A 4–8% difference in sequence will cause each human-specific 25-mer probe on a microarray to contain on average a single nucleotide mismatch with its target [32]. A large effect of sequence divergence on hybridisation signal was found in a study using a multi-primate cDNA array containing human, chimpanzee, orangutan and rhesus sequences, even between organisms that are only diverged ~1% [24]. The general picture that emerges is that the use of single species arrays for comparison between species may be problematic and yield spurious results [24], warranting careful data analysis and a significant amount of confirmatory analysis using other techniques. Meanwhile, the clear need for generation of species-specific cDNA and oligonucleotide-based microarray resources remains [32].

## Conclusion

In summary, we have generated the first marmoset-specific DNA microarray and demonstrated its use to characterise large scale gene expression profiles of hippocampus but also of other neuronal and peripheral tissues. In addition, we have generated a large collection of ESTs of marmoset origin which are now available in the public domain. These new tools will facilitate molecular genetic research into this non-human primate animal model.

## Methods

### Generation of marmoset cDNA sequences

Total RNA was isolated from hippocampi of 5 adult marmosets, both males and females ranging from 3 to 10 years in age, After checking the quality of the isolated RNA it was pooled and used to construct a custom normalised cDNA library in pCMVSport6.1 (Invitrogen, Carlsbad, USA). For the development of the marmoset microarray a total of 23,300 clones were gridded in 61 384-well plates. In total approximately 5700 sequencing reactions were performed at Baseclear (Leiden, The Netherlands) using an anchored oligo-dT primer on 15 randomly selected 384-well plates to generate partial 3' EST sequences.

### RNA preparation and labelling

Total RNA was isolated using TRIzol^® ^reagent (Invitrogen Life Technologies, Carlsbad, CA, USA) according to the manufacturer's instructions. After isolation, total RNA was purified using the QIAGEN RNEasy^® ^Mini Kit RNA Cleanup procedure (QIAGEN Inc., Valencia, CA, USA). RNA quality was assessed with the LabChip^® ^RNA 6000 Nano Assay on the 2100 Bioanalyzer (Agilent Technologies, Palo Alto, CA, USA). Per RNA sample, 5 μg was used as input into the MessageAmp™ II-Biotin *Enhanced *Single Round aRNA Amplification Kit (AM1791, Ambion, Austin, USA) according to the manufacturer's instructions. Briefly, total RNA was converted to double-stranded cDNA followed by in vitro transcription to synthesize biotin-labeled aRNA. After purification the size distribution of the aRNA was checked with the LabChip^® ^RNA 6000 Nano Assay on the 2100 Bioanalyzer (Agilent Technologies) and the quantity was assessed by small sample spectophotometry on a Nanodrop ND-1000 Spectrophotometer (NanoDrop Technologies, Wilmington, Delaware USA).

### Hybridisations

The biotinylated aRNA was fragmented and 15 μg was hybridised to marmoset arrays at the Leiden Genome Technology Center (LGTC^®^), Leiden University, The Netherlands, according to the manufacturer's recommendations (Affymetrix, Santa Clara, USA).

### Data analysis

Affymetrix GeneChip^® ^Operating Software (GCOS) was used to estimate signal intensities plus signal reliabilities. Data was median normalized in excel and subsequently scatterplots were constructed and correlation coefficients calculated using R version 2.2.0 [33]. Probe sets assigned with a gene symbol were functionally classified according to biological process and molecular function using Netaffx Analysis Center [34].

### Mapping and annotation of EST sequences

In order to obtain high-quality data the EST sequences were trimmed before further analysis. At the 3' end, at least 15% of each sequence was removed so that final DNA fragment lengths were 700 bases. In addition, 20 bases from the 5' end of the EST sequences were trimmed. For the annotation of the EST dataset, the sequences were reverse complemented and compared to the RefSeq dataset of human genes (NCBI Refseq build 36.1) using a locally installed version of the Blat software [35]. Remaining sequences that could not be unambiguously assigned to a gene name were mapped to the human genome sequence (NCBI Refseq assembly version 36.1) according to the standard parameters of the software. ESTs with a hit on the genome were annotated with the gene name of the gene which is located at the respective chromosome region. Ambiguities, e.g. if an EST sequence covered more than one gene, were resolved manually. Finally, a Blat search with increasing sensitivity parameters was performed to map ESTs across species.

The annotation procedure described above allowed a reliable annotation of EST data even if sequencing errors would cause frame-shifts in coding regions. In order to annotate open reading frames (ORFs) associated with the ESTs, ORFs with a length of at least 100 bases starting from the 5' end of the reverse complementary sequences were extracted. The translated amino acid sequences were mapped against the human protein sequences from UniProt (Release 50 of May 2006, [36]) using Blat [35]. All sequences without any hit on a protein sequence starting at the first position ofthe 5' end where rejected. The remaining candidate ORFs were first validated by a comparison of the gene names from the UniProt hits to the gene names of the above determined RefSeq hits. To be included as bona fide ORFs, the translated ORF sequences had to fulfil an additional criterion. The length of the peptide sequences derived from the ESTs had to cover at least 80% of the peptide sequence length which can be expected from the corresponding part of the protein sequence from UniProt.

## Authors' contributions

NAD generated the microarray, from sequence generation to design, coordinated the study and drafted the manuscript. MM carried out the RNA isolation, labeling and data analysis. SA and VWA contributed the marmoset sequences for CAT, EF2, GCLM, GPx1, GPx4, GSR and GSS and assisted with the initial gene selection. HZ isolated the hippocampal RNA used for the cDNA library and assisted in selection of marmoset housekeeping genes. CS was responsible for isolation and shipment of all marmoset tissues. BED and MAH performed the initial mapping and annotation of the sequences in 2004. BW and AR reannotated and performed the mapping of the sequences in 2006 and were responsible for GenBank submission. ERdK and EF had an advisory role in all steps of the array generation and validation. All authors read and approved the final manuscript.

## Supplementary Material

Additional file 1 **Gene content of the marmoset microarray and Gene Ontology Classification**. Excel file on content of the marmoset array. From left to right are columns indicating the Probe set ID on the marmoset microarray, the corresponding gene symbol (if known) and gene symbol aliases as well as the gene title. In the next column the source of the sequence is indicated, meaning whether it is derived from the set of 3215 ESTs or an already existing GenBank accession or if is a control sequence. Subsequently the GenBank accessions of the marmoset sequences and the gene ontology classification according to biological process, molecular function and cellular compartment, pathway and if applicable a pathway hyperlink are indicated. Click here for file

Additional file 3 **Overview of tissue-specific expression profiles**. Excel file showing hybridisation signals and detection calls for all tissues tested on the array. From left to right the Probe set ID on the marmoset array, the corresponding gene symbol (if known) and gene symbol aliases as well as the gene title are indicated. In the next columns the signals and the corresponding detection calls are listed for each tissues and finally a string of all detection calls is listed to facilitate identification of transcripts with a tissue-specific pattern of expression. Click here for file

Additional file 2 **Overview of 3215 sequences submitted to GenBank and dbEST**. Excel file giving a detailed overview of the panel of 3215 marmoset ESTs. From left to right the columns indicate the name of the EST clone, the corresponding gene symbol and aliases. The column "source" indicates if the sequence was mappable to a genome, could be assigned a gene name or not or contained a (partial) ORF. The next columns indicate the GenBank Accession number and whether the sequence was submitted to GenBank of dbEST. Finally, the last 2 columns indicate whether the sequence is represented on the marmoset array and if so by which probe set(s). Click here for file
